# Mechanism of Synergy between Piceatannol and Ciprofloxacin against *Staphylococcus aureus*

**DOI:** 10.3390/ijms232315341

**Published:** 2022-12-05

**Authors:** Mengyan Shi, Yubin Bai, Yanhua Qiu, Xinxin Zhang, Zikang Zeng, Lingling Chen, Fusheng Cheng, Jiyu Zhang

**Affiliations:** 1 Key Laboratory of New Animal Drug Project of Gansu Province, Lanzhou 730050, China; 2 Key Laboratory of Veterinary Pharmaceutical Development, Ministry of Agriculture, Lanzhou 730050, China; 3 Lanzhou Institute of Husbandry and Pharmaceutical Sciences, Chinese Academy of Agricultural Sciences, Lanzhou 730050, China

**Keywords:** piceatannol, ciprofloxacin, synergy, *S. aureus*, proton motive force

## Abstract

Piceatannol (PIC) is a natural stilbene extracted from grape skins that exhibits biological activities such as antibacterial, antitumor, and antioxidant activities. The present study was carried out to further investigate the effect of PIC on the antibacterial activity of different antibiotics and to reveal the antibacterial mechanism of PIC. We found that PIC had an inhibitory effect against *Staphylococcus aureus* (*S. aureus*); its minimum inhibitory concentration (MIC) and minimum bactericidal concentration (MBC) were 128 μg/mL and 256 μg/ mL, respectively. Additionally, we measured the fractional inhibitory concentration (FIC) of PIC combined with antibiotics via the checkerboard method. The results showed that when PIC and ciprofloxacin (CIP) were combined, they displayed a synergistic effect against *S. aureus*. Moreover, this synergistic effect was verified by time–kill assays. Further, the results of the membrane permeability assay and proton motive force assay revealed that PIC could enhance the sensitivity of *S. aureus* to CIP by dissipating the bacterial proton motive force (PMF), particularly the ∆ψ component, rather than increasing membrane permeability. PIC also inhibited bacterial adenosine triphosphate (ATP) synthesis and was less likely to induce bacterial resistance but exhibited slight hemolytic activity on mammalian erythrocytes. In summary, the combination of PIC and CIP is expected to become a new drug combination to combat *S. aureus*.

## 1. Introduction

*Staphylococcus aureus* (*S. aureus*) is a common Gram-positive bacterial pathogen that destroys host cells by adhering to host tissues, secreting extracellular toxins and enzymes. It can cause blood, skin, soft tissue, and upper respiratory tract infections, seriously endangering human and animal health [1,2]. According to reports, approximately 20,000 deaths happen annually from *S. aureus* infections in the United States [2]. Furthermore, in New Zealand, inpatients with *S. aureus* skin infections have risen from 0.81% in 2000 to 1.4% in 2011 [3]. In addition, *S. aureus* infection also causes substantial economic losses. According to statistics, the annual financial losses owing to mastitis in dairy cattle in Canada and the United States are USD318 million and USD2 billion, respectively [4].

Due to the abuse of antibiotics, several drug-resistant *S. aureus* strains have emerged worldwide, and roughly 90% of *S. aureus* strains are multi-drug-resistant bacteria [2,5]. Since the discovery of methicillin-resistant *Staphylococcus aureus* (MRSA) in the 1960s, the epidemic of MRSA has increased in Asia, Europe, and America [1]. The World Health Organization estimated that the number of deaths caused by drug-resistant bacterial infection is increasing yearly, and this number could probably rise to 10 million by 2050 [6]. Ciprofloxacin (CIP) belongs to the second generation of fluoroquinolone antibiotics, which has been widely favored since its listing due to its broad-spectrum antibacterial activity [7]. However, the widespread use of CIP has caused varying degrees of bacterial resistance in many regions, which has become a challenge in treating bacterial infections. Thus, it is necessary to seek new strategies to combat bacterial resistance.

Numerous studies have demonstrated the excellent antibacterial activity of natural products, for example, alpha mangostin, against MRSA by causing membrane permeabilization and destroying biofilms [8]; licorice extracts exhibited anti-MRSA activity by inhibiting biofilm formation and reducing the expression of exotoxin-related genes [9]. Furthermore, some studies have reported that many lactobacilli and bifidobacteria strains such as *Lactobacillus reuteri*, *Propionibacterium acnes*, and *Lactobacillus paracasei* can inhibit the growth of *S.aureus* [10]. First, these bacteria can compete for nutrients with *S.aureus* to hinder its growth. Second, they release bacteriocin and organic acids that inhibit biofilm formation and bacterial toxin production. In addition to developing new antimicrobial agents, mining new drug combinations based on existing drugs is also a solution to combat bacterial resistance. Compared with developing new drugs, drug combinations can save much time and reduce the dosage of medicines, thus reducing the toxicity and side effects of drugs. There have been many studies about drug combinations. For example, phenethyl isothiocyanate can restore the sensitivity of MRSA to auranofin by disrupting the cellular structure, increasing reactive oxygen species production and inhibiting biofilm formation [11]. Aspartic acid and succinic acid enhance the effects of CIP on *Pseudomonas aeruginosa* by inducing cell death and blocking the expression of CIP resistance genes, respectively [12].

Piceatannol (PIC) is a natural polyphenol compound first found in grape skin and abundant in passion fruit [13]. Recently, PIC has attracted much attention from scientists because of its wide range of biological activities. It has been reported that PIC has anti-proliferative effects on various cancer cells and has the potential for treating hypercholesterolemia and atherosclerosis [14,15,16,17]. As the hydroxyl compound of resveratrol, PIC has a more potent antioxidant and cytoprotective capacity than resveratrol [18]. Moreover, PIC also exhibits anti-inflammatory, anti-adipogenesis, and anti-melanin production activities [19]. Most importantly, PIC can inhibit the biofilm of *Streptococcus mutants* and exhibits antibacterial activity against *S. aureus* [20,21].

In this study, we explored the anti-*S. aureus* activity of PIC, evaluated the interaction between PIC and antibiotics, verified the synergistic effect of PIC combined with CIP, and further revealed the mechanism of PIC that enhances bacterial susceptibility to CIP against *S. aureus*.

## 2. Results

### 2.1. Antibacterial Activity of PIC

The MIC results of PIC against six *S. aureus* strains are shown in Table 1. The MIC of PIC was 64 μg/mL for J-6 and J-11 and 128 μg/mL for the other four strains. The results of MBC and the ratio of MBC to MIC for PIC against *S. aureus* are shown in Table 2. The results showed that PIC could kill five of the *S. aureus* strains with a concentration of 256 μg/mL, and the MBC/MIC ratios of the five strains did not exceed a value of four. The antibacterial activity showed no significant difference between CIP-sensitive strains and resistant strains.

### 2.2. The Combination of PIC and CIP Has a Synergic Effect

Subsequently, we determined the interaction between combinations of PIC and CIP, methicillin (MET), vancomycin (VAN), cefotaxime (CTX), tetracycline (TCY), and gentamicin (GEN). As shown in Table 3, combining PIC with GEN, VAN, CTX, and MET had additive effects on most of the *S. aureus* strains. PIC in combination with CIP had an additive effect on the ATCC29213, J-6, and J-9 strains and a synergistic effect on the J-28, J-11, and J-14 strains.

### 2.3. The Combination of PIC and CIP Shows Potently Bactericidal Activity against S. aureus

**Figure 1:**
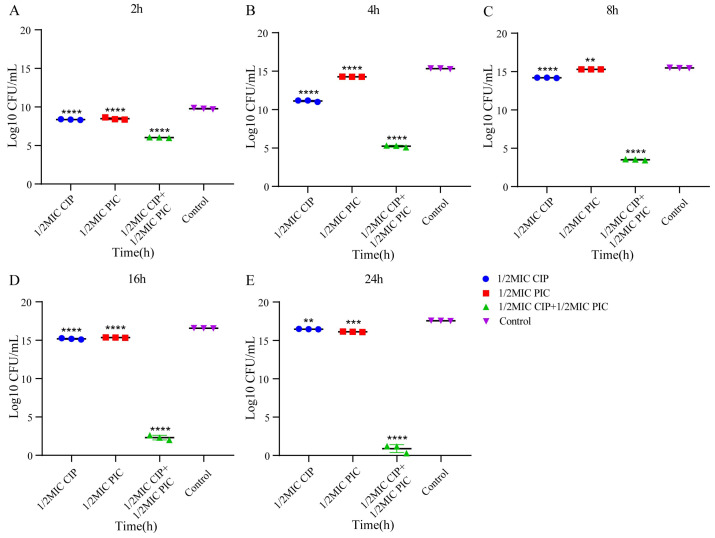
Statistical analysis of bacteria colonies of *S. aureus* J-14 at different times. (**A**) 2 h, (**B**) 4 h, (**C**) 8 h, (**D**) 16 h, (**E**) 24 h. n = 3. Results from all experiments are presented as the mean ± SD of three replicates. ** = *p* < 0.01, *** = *p* < 0.001, **** = *p* < 0.0001.

**Figure 2:**
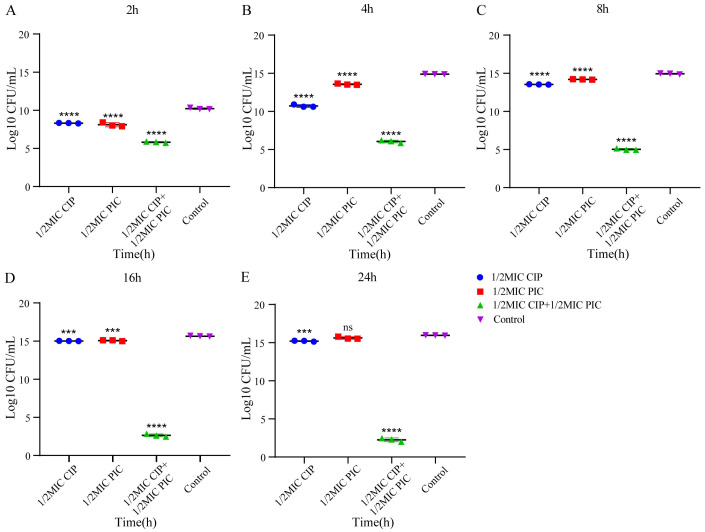
Statistical analysis of bacteria colonies of *S. aureus* ATCC 29213 at different times. (**A**) 2 h, (**B**) 4 h, (**C**) 8 h, (**D**) 16 h, (**E**) 24 h. n = 3. Results from all experiments are presented as the mean ± SD of three replicates. ns= *p* > 0.05, ***= *p* < 0.001, ****= *p* < 0.0001.

To further verify the synergic effect of PIC and CIP, the time–kill curves of PIC and CIP against *S. aureus* ATCC 29213 and the CIP-resistant strain J-14 were drawn, and the results were statistically analyzed. As shown in Figure 1 and Figure 2, the results showed that the number of colonies in all the test groups was highly significantly different (*p* < 0.0001) compared to the number of colonies in the control group at 2 h for these two strains. For strain J-14, the PIC group with 1/2MIC was significantly different (*p* < 0.01) at 8 h. Until 24 h, the 1/2MIC CIP group’s and the 1/2MIC PIC group’s statistical differences were significantly different (*p* < 0.01) and highly significantly different (*p* < 0.001), respectively. For strain ATCC 29213, the differences between 1/2MIC CIP and 1/2MIC PIC groups with the control group were both reduced from *p* < 0.0001 to *p* < 0.001 at 16 h. Finally, there was no statistical difference for the 1/2MIC PIC group when compared with the control group. Only the experimental group of combined CIP with PIC maintained a highly significant difference (*p* < 0.0001) within 24 h for these two strains.

**Figure 3:**
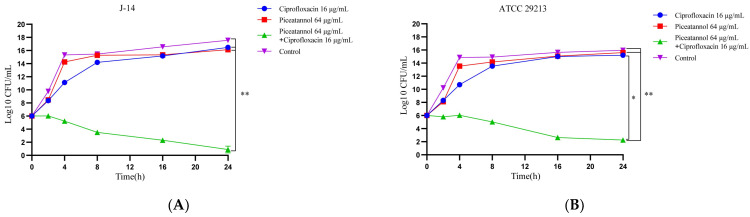
Time–kill curve of PIC and CIP against *S. aureus* strains. (**A**) J-14, (**B**) ATCC 29213. n = 3. Results from all experiments are presented as the mean ± SD of three replicates. * = *p* < 0.05, ** = *p* < 0.01.

As shown in Figure 3, in general, for *S. aureus* J-14, the combination group was significantly different (*p* < 0.01) compared with the other groups. A long-lasting bactericidal effect treated with CIP combined with PIC appeared at the beginning of treatment, with bacteria decreasing from 10^6^ CFU/mL to 10^3^ CFU/mL at 8 h, eventually reducing to about 10 CFU/mL. Nevertheless, for *S. aureus* ATCC 29213, the combination group was significantly different (*p* < 0.01) compared with the 1/2MIC PIC group and the control group and had statistical difference (*p* < 0.05) when compared with the 1/2MIC CIP group. The bacterial number in the combination group had decreased to about 10^5^ CFU/mL after 8 h, and 10^2^ CFU/mL of bacteria were left after 24 h. The results showed that combining PIC and CIP greatly enhanced antibacterial activity, and this gain effect was more significant against CIP-resistant strains.

### 2.4. PIC Dissipates the Proton Motive Force but Does Not Increase the Membrane Permeability of S. aureus

**Figure 4:**
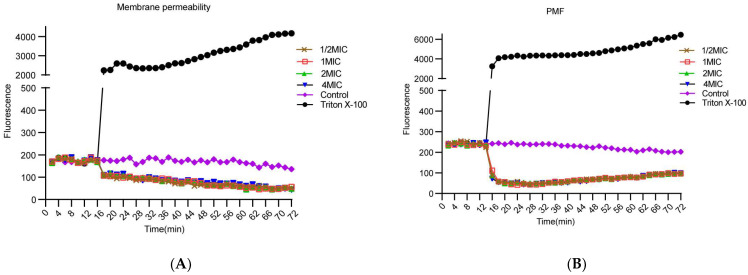
PIC on the proton motive force and the membrane permeability of *S. aureus* ATCC 29213. (**A**) Dynamic fluorescence of PI-treated *S. aureus* ATCC 29213 following the addition of PIC. (**B**) Dynamic fluorescence of Disc3(5)-treated *S. aureus* ATCC 29213 following the addition of PIC. n = 3. Results from all experiments are presented as the mean ± SD of three replicates.

Further, we investigated the antibacterial mechanism of PIC. Firstly, we conducted a membrane permeability assay (Figure 4A). The fluorescence value of PI did not increase at concentrations of 64 μg/mL, 128 μg/mL, 256 μg/mL, and 512 μg/mL of PIC. On the contrary, the fluorescence value slightly decreased, and there was no correlation between the change in fluorescence value and PIC concentration. This result indicates that PIC did not increase the permeability of the *S. aureus* cell membrane.

Next, we studied the change in bacterial PMF by potentiometric fluorophore 3, 3 ‘- dipropylthiadicarbocyanine iodide [Disc3(5)] (Figure 4B). The results showed that PIC caused a rapid decrease and then a slow rise in the fluorescence of Disc3(5). However, the concentration of PIC did not affect the change rate and degree of fluorescence value. This result indicates that PIC did not increase membrane permeability but exerted an anti-*S. aureus* effect through the dissipation of PMF, which was not concentration-dependent. This result is consistent with the results of the membrane permeability assay.

**Figure 5:**
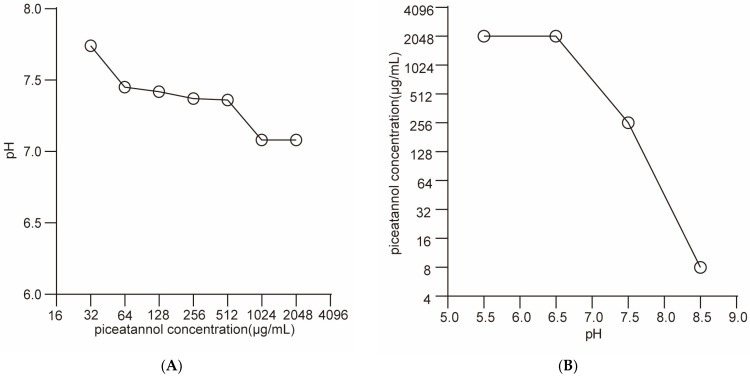
Effect of pH on the ability of PIC to dissipate *S. aureus* ATCC 29213 PMF. (**A**) pH of PBS with different concentrations of PIC. (**B**) MBC of PIC against *S. aureus* ATCC 29213 at different pH. n = 3. Results from all experiments are presented as the mean ± SD of three replicates.

Furthermore, we changed the dominant role of ∆ψ and ∆pH on PMF by changing the pH value of the medium to study the effects of PIC on ∆ψ and ∆pH (Figure 5). The results showed that the change in PIC concentration had little effect on pH. Then, by measuring MBC in different pH conditions, we found that MBC increased 8-fold in the acidic conditions and decreased 32-fold in the alkaline condition. These results demonstrate that the antibacterial activity of PIC significantly decreased when Δψ played a dominant role. This indicates that the destruction of PMF by PIC mainly depends on the dissipation of the Δψ component.

### 2.5. PIC Reduces the ATP Synthesis of S. aureus

**Figure 6:**
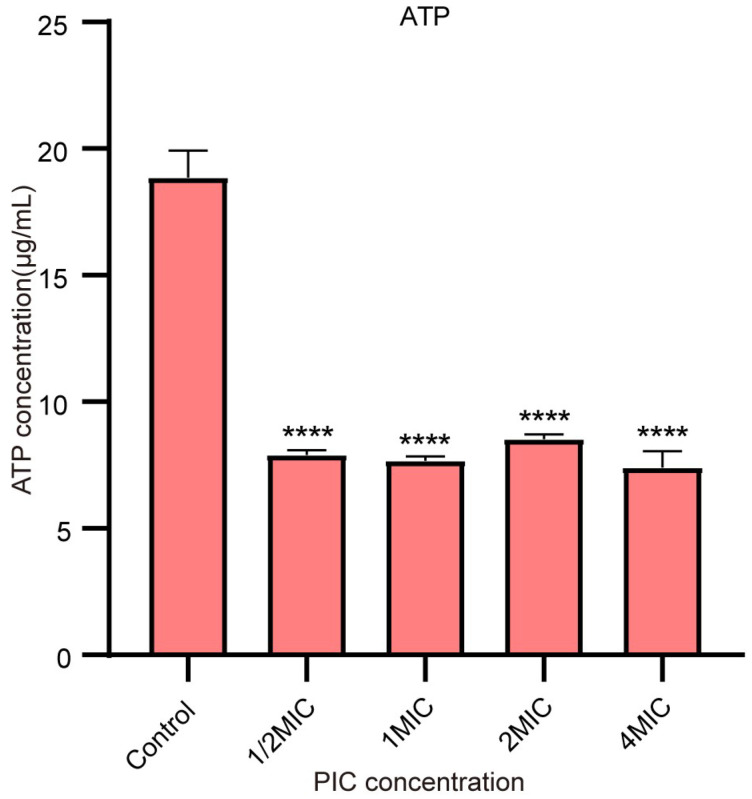
Intracellular ATP content of *S. aureus* treated with different concentrations of PIC. n = 3. Results from all experiments are presented as the mean ± SD of three replicates. **** = *p* < 0.0001.

The results of the ATP assay are shown in Figure 6. Compared with the control group, ATP content decreased by 41.9%, 40.6%, 45.3%, and 39.3% after 4 hours of 64 μg/mL, 128 μg/mL, 256 μg/mL, and 512 μg/mL PIC treatment, respectively. The results showed that the ATP content difference between the treatment group and the control group was highly significant (*p* < 0.0001). However, there were no significant differences among the four treatment groups. This result further proved that PIC dissipated the PMF of bacteria and affected the synthesis of ATP.

### 2.6. PIC Displays Low Levels of Resistance Development

**Figure 7:**
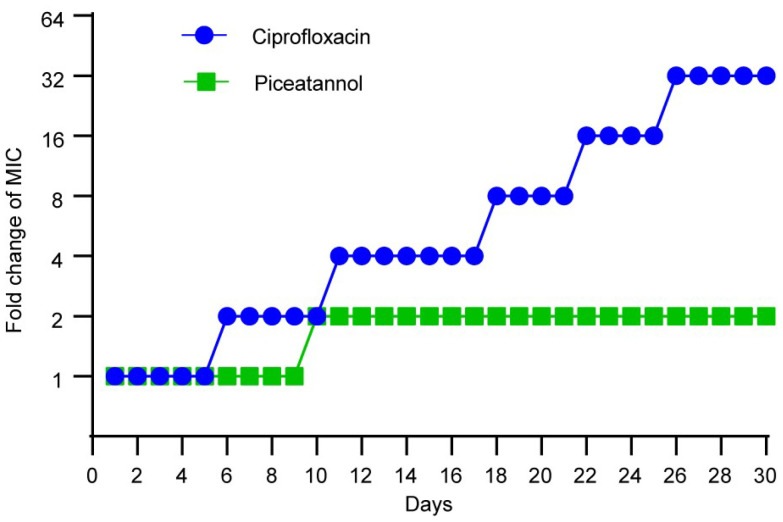
Resistance selection studies of PIC against *S. aureus* ATCC 29213. n = 3. Results from all experiments are presented as the mean ± SD of three replicates.

A resistance induction test was conducted in vitro to compare PIC with CIP to verify whether bacteria are prone to develop resistance to PIC. As shown in Figure 7, the antibacterial activity of CIP decreased 32-fold and that of PIC decreased only 2-fold during this experiment. The results indicated that bacteria do not easily develop resistance to PIC.

### 2.7. PIC has Slight Hemolytic Activity on Mammalian Erythrocytes

**Figure 8:**
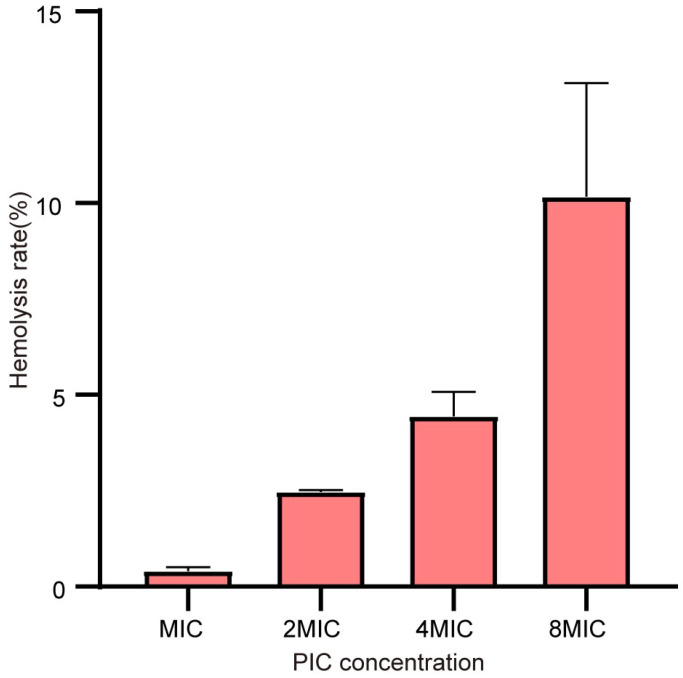
Hemolysis rate of sheep erythrocytes with different concentrations of PIC. n = 3. Results from all experiments are presented as the mean ± SD of three replicates.

To preliminarily evaluate the safety of PIC, its hemolytic activity on mammalian erythrocytes was measured. As shown in Figure 8, the hemolysis rate was about 10.2% with a concentration of 1024 μg/ mL. However, the hemolysis rate was only about 0.4% with the same concentration of MIC, and there was no hemolytic activity at lower concentrations. The results showed that PIC proved to be relatively safe to use in a specific concentration range.

## 3. Discussion

In recent years, natural products have shown great potential with regard to antibacterial, anti-oxidation, and anti-tumor activity [22]. An increasing number of studies have demonstrated that PIC exhibits excellent biological activity, such as anti-inflammatory and anti-tumor activity [16,17,23,24], and is expected to be a natural product that solves many medical problems in the future. However, studies on PIC’s antibacterial activity and mechanism are scarce. In this study, we found that PIC exhibits anti-*S. aureus* activity. Moreover, PIC could improve sensitivity to CIP and have a synergistic effect with CIP against *S. aureus*. PIC also inhibits the growth of *S. aureus* by dissipating PMF and inhibiting cellular ATP synthesis. Additionally, PIC displays low levels of resistance development and slight hemolytic activity. These results suggest that PIC is a membrane-active compound that acts against *S. aureus*.

By measuring the susceptibility of *S. aureus* to PIC and several antibiotics, we found that J-28, J-11, J-14, and J-9 were CIP-resistant strains, J-28 and J-9 were tetracycline-resistant strains, and J-28 and J-14 were also resistant to gentamicin. Among them, J-28 was a multi-drug-resistant bacterium. In addition, we determined the MIC of PIC against *S. aureus* to be 64∼128 μg/mL, which is different from that of a previous study [21]. In analyzing the results of the MIC and MBC, we found no difference in the antibacterial activity of PIC against drug-resistant and sensitive bacteria. Next, we calculated the MBC/MIC ratio to determine which ratio exhibited the antimicrobial effect of PIC against *S. aureus.* If MBC/MIC ≤ 4, the antimicrobial agent was defined to be bactericidal, but if MBC/MIC > 4, the antimicrobial agent was considered to be bacteriostatic [25]. The MBC/MIC ratios of five *S. aureus* strains were not higher than 4; thus, we presumed that the antibacterial effect of PIC against *S. aureus* was considered as the bactericidal effect. The results of antibiotic synergism tests showed that PIC could display a synergistic effect only when combined with CIP. Interestingly, the three *S. aureus* strains that exhibited synergism were all CIP-resistant bacteria, showing that the synergistic effect of PIC and CIP might be more substantial on CIP-resistant strains.

The results of the time–kill assay further confirm this synergistic effect. Based on the statistical analysis of the number of colonies of *S. aureus* J-14 and *S. aureus* ATCC 29213 at different times, we can see that CIP combined with PIC continuously maintained a strong bactericidal effect within 24 h. However, when CIP (1/2MIC) and PIC (1/2MIC) were used alone, the inhibitory effect decreased to different degrees after 8 h, especially for *S. aureus* ATCC 29213, and the number of colonies of the 1/2MIC PIC group had no difference when compared with that of the control group. These results indicate that the combination of CIP and PIC had a sustained killing effect on bacteria within 24 h and suggest that the antibacterial effect of the single-drug group would decrease over time.

Several membrane-active compounds exert their antibacterial effects by damaging cell membranes, such as diclofenac [26]. Others, such as daptomycin [27], do so by permeabilizing and depolarizing the cytoplasm. PI staining has always been considered the gold standard for determining membrane permeability; thus, we used this method to investigate whether PIC increased the membrane permeability of *S. aureus*. PI is a fluorescent dye with a high affinity to DNA. When the bacterial cell membrane is damaged, PI enters the cell and binds with DNA, and the fluorescence value increases rapidly [27]. However, the fluorescence value does not increase for bacteria with no membrane damage. Triton X-100 is a nonionic surfactant with a strong destructive effect on the cell membrane. It was used as a positive control to observe the increase in fluorescence value. In the present study, we found that PIC did not increase the PI fluorescence value but decreased it slowly. This result suggests that PIC did not act against bacteria by inducing membrane damage.

PMF is crucial to the survival of bacteria and consists of two main components: ∆pH formed by the pH difference value between intracellular and extracellular; and electric potential ∆ψ. Bacteria keep PMF constant by precisely regulating ∆ψ and ∆pH. The dissipation of any part of it will disturb the balance of the PMF of bacteria. However, PMF has been ignored as a target for antibacterial agents to a sizeable extent [5]. DiSC3(5) is a kind of fluorescent dye; changes in its fluorescence value can reflect changes in the PMF components ∆ψ and ∆pH of the cell membrane [6,12]. Fluorescence enhancement has shown increased cell membrane permeability or the ∆ψ dissipation of PMF. If the fluorescence value decreases, this indicates the dissipation of the ∆pH component. In this study, we found that PIC caused a rapid decrease in fluorescence followed by a slow increase, suggesting that PIC may affect both ∆ψ and ∆pH.

In proton dynamics, the dominant effects of ∆ψ and ∆pH are closely related to the pH of the environment [28]. ∆pH becomes the dominant component in an alkaline environment, and ∆ψ becomes the dominant component in an acidic environment. We determined the MBC of *S. aureus* ATCC 29213 in different acid–base backgrounds to explore which component was mainly perturbed by PIC. Our results showed that when ∆pH dominated, the anti-*S. aureus* activity of PIC was enhanced; when ∆ψ dominated, the antimicrobial activity was significantly reduced, indicating that when ∆ψ was dominant, PIC could not perturb enough ∆ψ to eradicate the PMF balance of bacteria. This result proves that PIC mainly perturbs the ∆ψ component of PMF to exert an anti- *S. aureus* effect. There are some compounds that have a similar effect to PIC; according to reports, the natural flavones morin and kuwanon G from morus alba [29] dissipated both the ∆ψ and ∆pH of the PMF of *S. aureus.* Furthermore, Ruhr and Sahl [30,31] found that the bacteriocin nisin produced by some *Lactococcus lactis* strains dissipates the ∆ψ of several Gram-positive bacteria.

The synthesis of ATP in cells is closely related to PMF. When PMF is destroyed, the synthesis of ATP is often affected [8]. The intracellular ATP level of *S. aureus* treated with PIC was detected. After 4 h, the intracellular ATP level was enormously decreased, proving that PIC can inhibit the ATP synthesis of *S. aureus*. Some studies have reported that PIC can inhibit the activity of the F1 part of F_0_F_1_-ATPase to halt ATP production [11]. In this study, PMF dissipation is responsible for the reduction in *S. aureus* ATP production. However, more research is needed to determine whether PIC can inhibit ATP production in *S. aureus* by targeting ATP synthase.

CIP belongs to a group of rapidly bactericidal antibiotics [32]. Based on the MBC/MIC ratio, PIC is also a bactericide. In addition, our study showed that PIC inhibits bacterial ATP synthesis, while CIP inhibits bacterial DNA synthesis to kill bacteria and prevent bacterial breeding [32]. Therefore, we hypothesize that the synergistic antibacterial effect of CIP and PIC is due to CIP making a further contribution to inhibiting genes involved in ATP synthesis.

The hemolysis assay is part of evaluating a compound’s safety in mammals. The hemolysis reaction is an adverse drug reaction wherein the compounds directly interact with erythrocytic elements or induce the body’s immune response leading to erythrocyte lysis [33]. Severe hemolysis reactions can lead to anemia, which is harmful to the health of the body. Many drugs have been reported to exhibit hemolytic activity, such as some nitrofurans and sulfonamides [33]. Thus, the hemolysis assay is important for the safety evaluation of drugs. The determination of the hemolytic activity of compounds with different concentrations can guide their rational dosage, so as to avoid the hemolysis reaction. PIC exhibits hemolytic activity on sheep red blood cells, and the hemolysis rate increases with the increase in concentration. These results suggest that the dosage of PIC should be strictly controlled to avoid systemic administration. Other safety evaluations need to be further conducted.

## 4. Materials and Methods

### 4.1. Materials and Bacterial Strains

*S. aureus* ATCC 29213 was purchased from the American Type Culture Collection. Clinical *S. aureus* strains J-14, J-11, J-6, J-9, and J-28 were isolated and preserved in our laboratory. Tryptic Soy Broth (TSB, Qingdao Hope Bio-Technology Co., Ltd., Qingdao, China) and Tryptic Soy Agar (TSA, Qingdao Hope Bio-Technology Co., Ltd., Qingdao, China) medium were used to cultivate all *S. aureus* strains. PIC and VAN were purchased from Shanghai Macklin Biochemical Co., Ltd., (Shanghai, China). GEN, TCY, CTX, CIP, and MET were purchased from Beijing Solarbio Science & Technology Co., Ltd., (Beijing, China). PIC was dissolved in dimethyl sulfoxide (DMSO). CIP and VAN were dissolved in sodium hydroxide solution. GEN, TCY, and CTX were dissolved in the TSB medium.

### 4.2. Antimicrobial Activity

#### 4.2.1. Determination of Minimum Inhibitory Concentration (MIC) and Minimum Bactericidal Concentration (MBC)

The MICs were determined using the broth microdilution method as described by the Clinical and Laboratory Standards Institute [34]. Bacteria were cultured in TSB medium at 37 °C to reach the logarithmic phase; then, we adjusted the cultured bacteria with TSB to 0.5 McFarland’s standard and diluted into TSB at a 1:100 dilution. Next, the compounds were prepared with TSB on 96-well plates by serial twofold dilutions. The growth control group and sterility control group were also studied for each strain. The diluted 100 μL bacterial solution was added to each well of the plates and incubated for 16–18 h at 37 °C. The MIC was defined as the lowest concentration that inhibited bacterial growth. The determination of MBC was performed using the method of previous studies [34,35]. A total of 100 μL of bacterial suspensions was taken from the wells of the MIC, 2MIC, 4MIC, and 8MIC experiments and spread on TSA plates, then incubated for 18–24 h at 37 °C. The number of bacteria colonies was counted and the minimum concentration that led to the complete absence of colony growth was defined as the MBC.

#### 4.2.2. Antibiotic Synergism Tests

The checkerboard method was used to determine the combined antibacterial effect of PIC and the six antibiotics [36,37]. A 0.5 McFarland’s standard was prepared by diluting the cultured bacteria into TSB at a 1:100 dilution. Antibiotics and PIC were twofold serially diluted in a 96-well plate; then, we added 100 μL of the bacterial solution to each well. Each experiment was checked in triplicate and repeated twice. The lowest concentration with no visible bacterial growth was recorded as the MIC combination. Finally, the fractional inhibitory concentration (FIC) was calculated according to the following formula to determine the interaction between PIC and antibiotics, where FIC ≤ 0.5, synergism; 0.5 < FIC ≤ 1, additive effect; 1 < FIC ≤ 2, no effect; FIC > 2, antagonistic effect:

(1) $$\mathrm{FIC}=\frac{A(\mathrm{MIC}\;\mathrm{of}\;\mathrm{combination})}{A(\mathrm{MIC}\;\mathrm{of}\;\mathrm{alone})}+\frac{B(\mathrm{MIC}\;\mathrm{of}\;\mathrm{combination})}{B(\mathrm{MIC}\;\mathrm{of}\;\mathrm{alone})}$$

#### 4.2.3. Time–Kill Assay

The time–kill assay was used to verify the synergistic effect of PIC combined with CIP [37]. *S. aureus* ATCC 29213 and J-14 at exponential phase were adjusted in TSB to obtain a bacterial suspension of about 1.0 × 106 CFU/mL and then treated with 1/2MIC PIC, 1/2MIC CIP, and 1/2MIC PIC + 1/2MIC CIP; the bacterial suspensions treated with sodium hydroxide solution and DMSO were incubated as control. The bacteria were removed from the cell cultures at 0 h, 2 h, 4 h, 8 h, 16 h, and 24 h, were serially diluted with TSB, and then each dilution was spotted on the TSA plate. After being incubated at 37 °C for 16–18 h, the colonies on the plate were counted.

### 4.3. The Antibacterial Mechanism

#### 4.3.1. Membrane Permeability Assay

The membrane permeability assay was used to observe the effect of *S. aureus* membrane permeability treated by PIC [27]. *S. aureus* ATCC 29213 was cultured in TSB at 37 °C for 16 h and washed three times with PBS. The cultures were adjusted to match the turbidity of the 0.5 McFarland turbidity standard, then incubated with propidium iodide (PI) with a concentration of 5 μg/mL for 30 min at 37 °C in the dark. After incubation, 50 μL cultures were added to each well on a black polystyrene microtiter plate. An automatic microplate reader was used to measure the fluorescence value for 535 nm wavelength excitation and 620 nm emission every 2 min for 15 min. Subsequently, the PIC was rapidly added to the micropores at final concentrations of 1/2MIC, MIC, 2MIC, and 4MIC, and the fluorescence value was measured every 2 min for 1 h. Solution 1% Triton X-100 was used as the positive control, and no drug was used as a negative control.

#### 4.3.2. Proton Motive Force Assay

The proton motive force assay was used to observe the effect of *S. aureus* PMF treated by PIC [29]. *S. aureus* ATCC 29213 was cultured in TSB at 37 °C for 16 h and centrifuged at 3000× *g* for 10 min. Afterward, the cells were washed three times with HEPES (containing 20 mM glucose, pH 7.2) and resuspended with HEPES. The cultures were adjusted to match the turbidity of the 0.5 McFarland turbidity standard with HEPES. Disc3(5) with a concentration of 1 μM was added into the bacterial suspensions and incubated for 15 min in the dark. After incubation, the cultures were quickly added to a black polystyrene microtiter plate, and the fluorescence value was measured for 660 nm wavelength excitation and 675 nm emission every 2 min for 15 min. Subsequently, the PIC was rapidly added, and the fluorescence value was measured every 2 min for 1 h. Solution 1% Triton X-100 was used as the positive control, and no drug was used as a negative control.

#### 4.3.3. Effects of pH on Antibacterial Activity

This assay was used to observe the effects of antibacterial activity with different pH values [28]. A certain concentration of PIC was dissolved in TSB to reach final concentrations of 2048 μg/mL, 1024 μg/mL, 512 μg/mL, 256 μg/mL, 128 μg/mL, 64 μg/mL, and 32 μg/mL, and the pH of the solution was determined with a pH meter (Thermo Fisher Technology (Shanghai, China) Co., Ltd.). Sodium carbonate and hydrochloric acid were used to adjust the medium’s pH to 5.5, 6.5, 7.5, and 8.5. Then, the MBC of *S. aureus* ATCC 29213 was determined using different pH media.

#### 4.3.4. ATP Assay

The ATP assay was used to explore the effect of *S. aureus* ATP concentration treated by different concentrations of PIC. *S. aureus* ATCC 29213 was cultured in TSB at 37 °C for 16 h. The experimental group was incubated with a MIC concentration of PIC for 4 h. After incubation, the bacterial suspensions were centrifuged at 3000× *g* for 10 min and washed three times with PBS. The bacterial suspensions were adjusted to 1.0 × 107 CFU/mL, and the supernatant was discarded by centrifugation. The samples without PIC were marked as control. ATP was determined according to the instructions of the ATP Assay Kit (Beyotime Biotechnology Co., Ltd., Shanghai, China).

### 4.4. Resistance Study

A resistance study was used to explore the susceptibility of PIC to inducing the development of *S. aureus* resistance [38]. *S. aureus* ATCC 29213 was cultured in TSB at 37 °C for 16 h, and the initial MICs of PIC and CIP were determined. Then, the bacteria were passaged to new TSB containing PIC and CIP with a concentration of 1/2MIC. The MIC was measured again with the cultured bacteria. This operation was repeated for 30 passages, and MIC changes were observed.

### 4.5. Hemolysis Assay

The hemolysis assay was used to study the hemolytic activity of PIC on mammalian erythrocytes [38]. Fresh sheep red blood cells were washed three times with PBS and then diluted with PBS to obtain an 8% red blood cell (*v/v*) solution. The diluted cells were added with different concentrations of PIC (1024 μg/mL, 512 μg/mL, 256 μg/mL, 128 μg/mL, and 64 μg/mL) and incubated at 37 °C for 1 h. Solution 0.2% Triton X-100 was used as the positive control, and no drug was used as a negative control. After incubation, the samples were centrifuged at 4000 rpm for 10 min. The supernatants were placed in a 96-well plate to measure absorbance at 576 nm. The hemolysis rate was calculated as:

(2) $$\mathrm{Hemolysis}\;\mathrm{rate}\left(\%\right)=\frac{\mathrm{Abs}\left(\mathrm{sample}\right)-\mathrm{Abs}\left(\mathrm{negative}\;\mathrm{control}\right)}{\mathrm{Abs}\left(\mathrm{positive}\;\mathrm{control}\right)-\mathrm{Abs}\left(\mathrm{negative}\;\mathrm{control}\right)}*100$$

### 4.6. Statistical Analysis

Experimental results were statistically analyzed by GraphPad Prism 8.0.1 (GraphPad Software, San Diego, CA, USA). Non-parametric one-way ANOVA was applied to determine the significant differences at a significance level of *p* < 0.05. The data were reported as means of three replicates ± standard deviation (SD).

## 5. Conclusions

In conclusion, the combination of PIC and CIP had a solid and long-lasting synergistic effect on both drug-resistant and susceptible strains of *S. aureus*. PIC dissipated the ∆ψ component of PMF, reduced the intracellular ATP production of *S. aureus*, and exhibited a relatively low level of resistance development. Additionally, when using PIC, we should prioritize topical medication and strictly control the dosage within 128 μg/mL to avoid hemolysis. In summary, our study combined PIC with antibiotics for the first time and found the effective drug combination of PIC with CIP, which suggests that in antimicrobial drug development, the focus could be on combining natural products with existing antibiotics, thus restoring bacterial susceptibility to antibiotics. This strategy will be effective in stopping the spread of bacterial resistance in the absence of novel antibiotics.

## Figures and Tables

**Table 1:** Minimum inhibitory concentrations of piceatannol and several antibiotics against *Staphylococcus aureus* (μg/mL).

Strain	PIC	CIP	MET	VAN	CTX	GEN	TCY
ATCC 29213	128	4	2	4	2	2	0.25
J-28	128	32	2	2	1	16	32
J-6	64	4	2	4	1	0.25	0.25
J-11	64	32	1	1	1	4	0.25
J-14	128	32	2	2	2	32	0.25
J-9	128	32	2	2	1	8	32

PIC: piceatannol, CIP: ciprofloxacin, MET: methicillin, VAN: vancomycin, CTX: cefotaxime, GEN: gentamicin, TCY: tetracycline.

**Table 2:** Minimum bactericidal concentrations and the ratio of minimum bactericidal concentration to minimum inhibitory concentration of piceatannol against different *Staphylococcus aureus* strains.

Strains	MBC (μg/mL)	MBC/MIC
ATCC 29213	256	2
J-28	256	2
J-6	512	8
J-11	256	4
J-14	256	2
J-9	256	2

**Table 3:** Fractional inhibitory concentration index of piceatannol combined with different antibiotics against *Staphylococcus aureus* strains.

Antibiotics	ATCC 29213	J-28	J-6	J-11	J-14	J-9
CIP	0.75	0.5	0.75	0.375	0.375	1
MET	2	0.75	0.75	1	1	0.75
VAN	1.25	1	0.5625	0.75	1	1
CTX	2.5	1	2	1	1	0.75
GEN	0.625	0.625	0.75	1.125	0.75	0.75
TCY	0.75	0.75	0.75	2	2	0.75

CIP: ciprofloxacin, MET: methicillin, VAN: vancomycin, CTX: cefotaxime, GEN: gentamicin, TCY: tetracycline.

## Data Availability

Not applicable.
